# Acupuncture for chronic insomnia disorder: a systematic review with meta-analysis and trial sequential analysis

**DOI:** 10.3389/fneur.2025.1541276

**Published:** 2025-04-30

**Authors:** Yi Yu, Xinju Li, Zheng Zhu, Yingdong Wang, Qiang Xi, Jiwen Qiu, Yidan Xu, Ruonan Liang, Yi Guo, Mingxing Zhang

**Affiliations:** ^1^School of Acupuncture & Moxibustion and Tuina, Tianjin University of Traditional Chinese Medicine, Tianjin, China; ^2^Research Center of Experimental Acupuncture Science, Tianjin University of Traditional Chinese Medicine, Tianjin, China; ^3^School of Traditional Chinese Medicine, Tianjin University of Traditional Chinese Medicine, Tianjin, China; ^4^School of Medical Technology, Tianjin University of Traditional Chinese Medicine, Tianjin, China; ^5^School of Intergrative Medicine, Tianjin University of Traditional Chinese Medicine, Tianjin, China

**Keywords:** acupuncture, chronic insomnia disorder, systematic review, meta-analysis, trial sequential analysis

## Abstract

**Objective:**

To investigate the effect of placebo response to acupuncture on subjective and objective sleep indices in patients with chronic insomnia disorder and to understand the effectiveness of acupuncture in the treatment of chronic insomnia disorder (CID).

**Methods:**

A comprehensive search was conducted from the inception of the databases to March 17, 2025, encompassing eight databases. A randomized controlled pilot study of collecting acupuncture versus sham acupuncture for the treatment of CID. Systematic collection of acupuncture therapies for CID was performed based on randomized controlled trials (RCTs). Independent researchers critically reviewed the literature, recorded relevant data, and assessed the quality of research. Data were analyzed using RevMan 5.3, Stata 17.0, and TSA 0.9.5.10.

**Results:**

The study included a total of 757 patients across 10 trials. Acupuncture demonstrated significant improvement in PSQI scores [MD = −2.60, 95% CI = (−3.24, −1.97), *p* < 0.00001] and ISI scores (MD = −2.04, 95% CI = [−3.18, −0.90], *p* = 0.0005) compared to sham acupuncture. Sequential analyses of the trials showed stable results. Subgroup analyses showed that manual acupuncture and electroacupuncture were superior to sham acupuncture in improving PSQI scores [MD = −3.85, 95% CI = (−4.94, −2.76), *p* < 0.00001; MD = −1.67, 95% CI = (−2.25, −1.08), *p* < 0.00001]. Manual acupuncture and electroacupuncture were superior to sham acupuncture in improving ISI scores [MD = −2.60, 95% CI = (−4.72, −0.48), *p* = 0.02; MD = −1.93, 95% CI = (−3.16, −0.71), *p* = 0.002]. In terms of objective sleep indices, there was no statistically significant difference in total sleep time between acupuncture and sham acupuncture [MD = 11.92, 95% CI = (−20.25, 44.09), *p* = 0.47]. Acupuncture was superior to sham acupuncture in terms of sleep efficiency and wake after sleep onset [MD = 3.62, 95% CI = (0.92, 6.32), *p* = 0.009; MD = −18.53, 95% CI = (−29.22, −7.85), *p* = 0.0007]. However, the sequential analysis indicated limitations due to small sample size which hindered drawing definitive conclusions.

**Conclusion:**

Compared with sham acupuncture, acupuncture is effective in improving subjective sleep quality in patients with CID. However, whether acupuncture improves patients’ objective sleep indices compared to sham acupuncture is uncertain and more high-quality clinical trial evidence is needed to validate this.

**Systematic review registration:**

https://www.crd.york.ac.uk/PROSPERO/, Identifier CRD42024541760.

## 1 Introduction

Chronic insomnia disorder (CID) is characterized by the inability to initiate or maintain sleep, as well as frequent nocturnal awakenings with difficulty returning to sleep. It commonly presents with dissatisfaction regarding both the duration and quality of sleep, leading to symptoms such as fatigue, depression, cognitive impairment, headaches, etc. These symptoms occur at least three times per week and persist for a minimum of 3 months (1). Within 1 year of diagnosis, approximately 86% of individuals with insomnia continue to experience symptoms, while more than half (59%) endure them for over 5 years (2). In a longitudinal study conducted in 2009, it was found that after 1 year, 181 out of 244 patients with insomnia still experienced symptoms of insomnia (3). Insomnia disorders lead to an increased risk of cardiovascular disease, obesity, depression, cognitive impairment, and severe insomnia may even lead to accidents (4). Research has demonstrated the substantial costs associated with insomnia, including direct treatment expenditures, reduced work efficiency, and increased accident risk (5). A Canadian study estimated that the economic burden of insomnia symptoms amounted to C$1.9 billion. A 5% increase in these symptoms would result in an estimated additional expenditure of $333 million per year (6). Both the healthcare system and patients bear a significant burden due to CID.

The treatment modalities for CID encompass both pharmacological and non-pharmacological treatments. Currently, pharmaceutical treatments comprise benzodiazepines, antihistamines, and melatonin agonists; however, sustained remission is not achieved in approximately 40 percent of patients (7). Moreover, there exists a risk of drug resistance, dependence, and abuse. Prolonged use of these medications may lead to drowsiness, fatigue, and sleepwalking (8). Among the non-pharmacological approaches recommended by clinical guidelines for CID management is multicomponent cognitive behavioral therapy (CBT), yet challenges such as intricate implementation procedures, high economic costs, and stringent therapist requirements persist (9, 10). Additionally, it should be noted that sleep restriction therapy within CBT-I is contraindicated for individuals engaged in high-risk occupations or those prone to mania or poorly controlled epilepsy (11).

Acupuncture has been utilized in China for numerous years as a therapeutic approach for CID. Clinical studies have demonstrated that acupuncture effectively increased sleep time, reduced sleep awakenings, improved sleep quality, and effectively improved anxiety, depression, and fatigue in patients with CID (12–14). Moreover, it is considered safe with no significant adverse effects. However, the limited sample size of randomized controlled trials (RCTs) investigating acupuncture treatment for CID restricts the strength of evidence provided. Meta-analyses of acupuncture versus sham acupuncture for the treatment of patients with insomnia disorders have come to contradictory conclusions, although both studies have shown that acupuncture significantly improves the subjective sleep quality of patients when compared to sham acupuncture. However, in terms of objective sleep indices, one study showed that acupuncture versus sham acupuncture increased total sleep time, improved sleep efficiency, and decreased awakenings after sleep onset (15); While another study found no statistically significant differences between acupuncture versus sham acupuncture in terms of improving total sleep time, sleep efficiency, and awakenings after sleep onset (16). Is this related to the failure to qualify the type of insomnia (primary vs. secondary insomnia) and the duration of the disease (chronic vs. non-chronic insomnia) and to standardize the inclusion of the assessment? Differences in the type and duration of insomnia require different acupuncture treatment modalities or cycles to achieve therapeutic efficacy, and acupuncture should be evaluated individually for the treatment of primary chronic insomnia disorders.

Consequently, this study aims to conduct a comprehensive meta-analysis utilizing published randomized sham controlled trial focused on CID in order to furnish substantial evidence supporting acupuncture as a viable treatment option. In addition, Trial Sequential Analysis (TSA) enhances the methodological robustness of meta-analyses by combining cumulative meta-analysis with sequential monitoring boundaries (17). Key strengths include quantifying evidence sufficiency through the required information size (RIS) to determine whether conclusions are stable or necessitate further studies. Meanwhile, TSA is essential in small-sample or high heterogeneous meta-analyses, addressing uncertainty in statistically significant outcomes and providing a quantitative framework for clinical decisions and trial design. Therefore, TSA is conducted to assess the robustness of the meta-analysis findings and determine the need for additional studies.

## 2 Methods

### 2.1 Search strategy

The search was conducted by two evaluators using the following databases: (1) PubMed, Embase, Cochrane Central Register of Controlled Trials database (CENTRAL) and Web of Science (WOS); and (2) China National Knowledge Infrastructure (CNKI), VIP Database, WF Database and SinoMed Database (CBM). We searched for studies published from the inception of the databases to March 17, 2025. A three-part search strategy was employed based on disease identification (CID), intervention analysis (acupuncture), and study design classification (RCT). Detailed search strategy is presented in Supplementary File 1. The program has been registered with PROSPERO. The registration number was CRD42024541760.

### 2.2 Inclusion criteria

#### 2.2.1 Participants

Met at least one of the following diagnostic criteria: (1) the Diagnostic and Statistical Manual of Mental Disorders, Fifth Edition (DSM-5) or Fourth Edition (DSM-IV); (2) the International Classification of Sleep Disorders, Third Edition (ICSD-3); (3) the Chinese Guidelines for Diagnosis and Treatment of Insomnia Disorders.

All participants fulfilled the diagnostic criteria for CID, with sleep disturbances occurring ≥3 times per week and persisting for ≥ 3 months. The study population was not restricted by sex, ethnicity, or nationality.

#### 2.2.2 Interventions

The experimental group was treated with acupuncture therapies such as manual acupuncture, electroacupuncture, and transcutaneous acupoint electrical stimulation. The intervention protocol included specifications of acupoint selection, needling techniques, stimulation parameters, treatment duration, and session frequency.

#### 2.2.3 Comparators

In the control group, sham acupuncture was administered using the following methods: (1) Piercing non-acupuncture points (located ≥1 cm from standard acupoints); (2) Contact with the skin without piercing the skin (e.g., Streitberger placebo needles); (3) Piercing points unrelated to the treatment of insomnia (e.g., Huantiao [GB30], which is primarily used for lower limb pain disorders). The duration and period of treatment in the control group were kept the same as in the experimental group.

#### 2.2.4 Outcomes

Outcome indicators included any of the following: (1) PSQI: Sleep quality was assessed using the Pittsburgh Sleep Quality Index (PSQI); (2) ISI: Insomnia severity was evaluated with the Insomnia Severity Index (ISI); (3) Objective sleep parameters were recorded via polysomnography (PSG) or actigraphy: sleep time (TST; in minutes), sleep efficiency (SE; TST/total recording time × 100%), and wake after sleep onset (WASO; total recording time - lights out to first epoch of any sleep - TST; in minutes).

#### 2.2.5 Research type

Randomized controlled trials (RCTs) were included. Studies with complete reporting of group sample sizes and outcome measures.

### 2.3 Exclusion criteria

(1) Studies involving acupuncture in combination with herbal medicine, western medicine or other interventions;
(2) Narcolepsy, respiratory-related sleep disorders, circadian rhythm sleep–wake disorders and parasomnias;
(3) Insomnia caused by other diseases, such as tumors and strokes;
(4) Duplicated publication;
(5) Studies lacking critical data that could not be extracted or accessed;
(6) Animal experiments.

### 2.4 Data extraction

The first author, year of publication, country of origin, participants’ characteristics (age and sex), intervention details, control group information, duration of the intervention, and outcome indicators were systematically tabulated. The selection and extraction of data were conducted independently by two researchers in accordance with the PRISMA guidelines (see Supplementary File 2). In case of any disagreement regarding data statistics, a third investigator was involved for assessment.

### 2.5 Study quality and risk of bias assessment

The risk of bias in the studies was independently assessed by two investigators using the Cochrane Collaboration’s risk of bias tool (ROB) in Revman 5.3 software (18). The assessment encompassed random sequence generation, allocation concealment, blinding, assessment of incomplete data, selective outcome reporting and other sources of bias. If there was disagreement between two investigators’ assessments, a third investigator performed the assessment. Each item was categorized as having low, high, or unclear risk of bias.

Standards for Reporting Interventions in Clinical Trials of Acupuncture (STRICTA) checklist (revised version, published 2010) for assessing the quality of clinical trial reports (19). The assessment included acupuncture rationale, details of needling, treatment regimen, other components of treatment, practitioner background and control or comparator interventions.

### 2.6 Data analysis

The meta-analysis was performed using Revman 5.3 software. Continuous variables were expressed as mean difference (MD), and effect sizes along with their corresponding 95% confidence intervals were calculated based on pre- and post-treatment differences. Heterogeneity of effect sizes was assessed using the I2 index. A fixed-effect model was employed when heterogeneity was low (I^2^ < 50%, *p* ≥ 0.1). In cases of high heterogeneity (I^2^ ≥ 50%, *p* < 0.1), a random-effects model was used, and sensitivity analyses were performed with Stata 17.0 software for further investigation. Additionally, funnel plots were generated once the number of included RCTs reached 10. Egger’s test was used to examine potential reporting bias in Stata 17.0.

Trial sequential analysis (TSA) was conducted using TSA 0.9.5.10 (http://www.ctu.dk; Copenhagen Trial Unit, Denmark). The sample size was used as the expected information value, and the analysis was performed with a significance level of *α* = 0.05 for type I error, power of *β* = 0.2 for type II error, and a statistical efficacy of 80% to minimize random errors and ascertain the reliability of the results. Additionally, an estimation of the required sample size for meta-analysis was also carried out.

## 3 Results

### 3.1 Study selection

**Figure 1:**
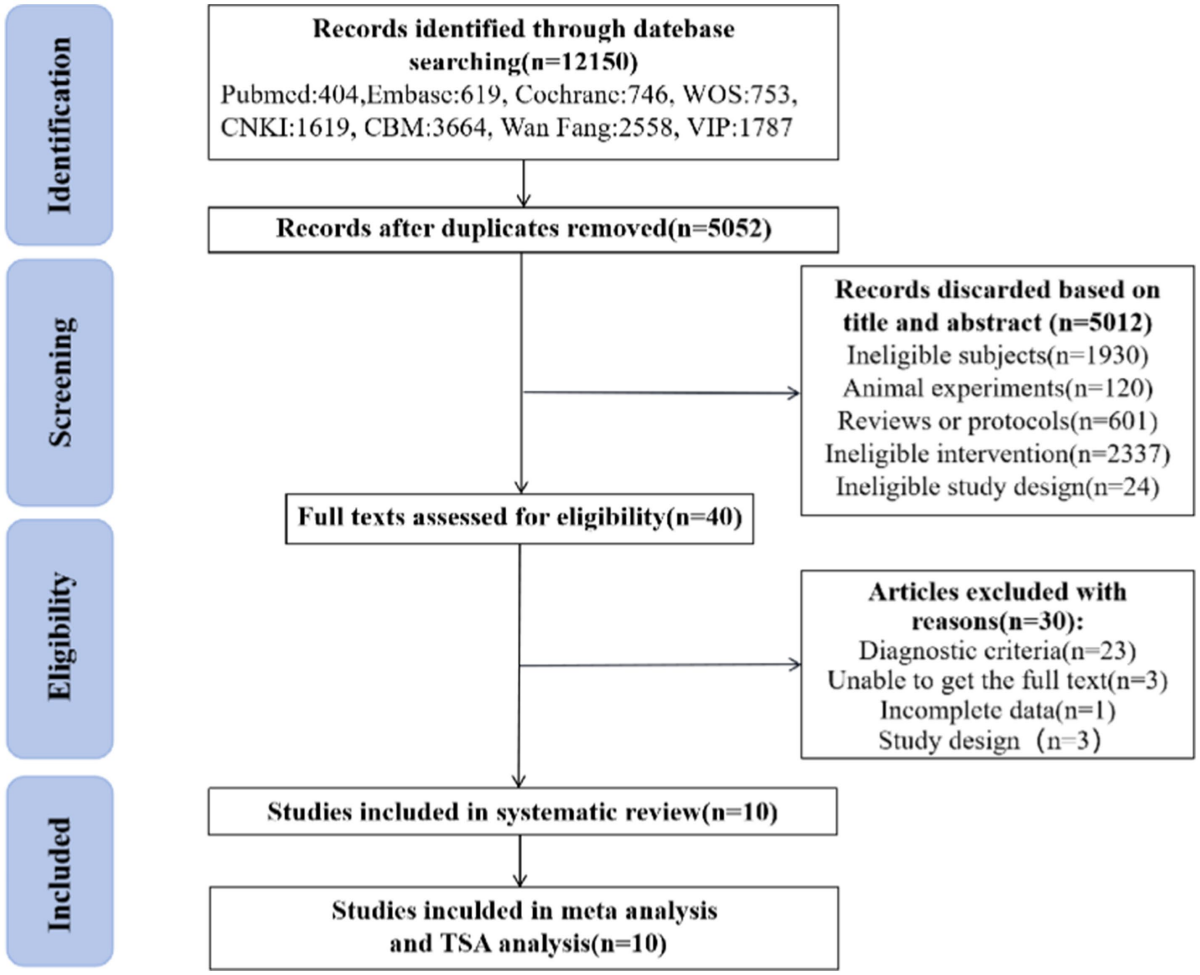
PRISMA flow diagram of included articles.

A total of 12,150 documents were retrieved and 7,098 duplicates were eliminated using Noteexpress software. Among the remaining 5,052 literatures, 5,012 were excluded from further consideration based on their titles and abstracts. Eventually, after reading through 40 full-text articles, a systematic review, meta-analysis and TSA analysis was conducted on a final selection of 10 literatures (12, 13, 20–27). The flowchart illustrating the literature screening process is presented in Figure 1.

### 3.2 Characteristics of included studies

This study encompassed 10 papers. Notably, the data from Lee1 2020 and Lee2 2020 were derived from a split of the same study, thus considered as one cohesive study (12). The collective sample size comprised 757 participants, with 376 assigned to the experimental group and 381 to the control group. Among these subjects, there were 229 males and 528 females. The included studies consisted of four conducted in English (12, 13, 21, 24) and six in Chinese (20, 22, 23, 25–27). Manual acupuncture was used in five studies (13, 23, 24, 26, 27), while electroacupuncture was used in five studies (12, 20–22, 25). Ten studies used PSQI to assess patients’ sleep quality. Two studies (13, 25) evaluated patients’ sleep quality using PSG, while one study evaluated patients’ sleep quality using ActiGraph (21). A comprehensive list of the included studies is provided in Table 1.

**Table 1:** Basic information of the included literature.

Study	Country	Sample size/case	Sex/Case (M: F)	Age/years (^−^*χ* ± s)	Intervention	Course	Outcome indicator
Lee et al. (12)	Korea	I: 49 C: 52	I: 9:40 C: 9:43	I: 51.78 ± 1.14 C: 52.00 ± 1.10	I: EA C: Sham EA (a blunt end not penetrating the skin at sham acupoints)	1: 2 wks 2: 4 wks	PSQI ISI
Wang et al. (13)	China	I: 41 C: 41	I: 13:28 C: 10:31	I: 55.32 ± 10.85 C: 56.22 ± 11.08	I: MA C: Sham MA (shallow needling of sham acupoints)	3.5 wks	PSQI ISI PSG
Xi et al. (20)	China	I: 29 C: 29	I: 12:17 C: 13:16	I: 44 ± 12 C: 41 ± 12	I: EA C: Sham EA (shallow needling of non-effective acupuncture)	4 wks	PSQI
Yeung et al. (21)	China	I: 30 C: 30	I: 8:22 C: 6:24	I: 48.3 ± 9.5 C: 47.8 ± 8.6	I: EA C: Sham EA (a blunt end not penetrating the skin at the same acupoints)	3 wks	PSQI ISI Actigraphy
Yuan et al. (22)	China	I: 30 C: 32	I: 20:10 C: 20:12	I: 45.93 ± 14.08 C: 45.84 ± 12.67	I: EA C: Sham EA (a blunt end not penetrating the skin at sham acupoints)	2 wks	PSQI ISI
Zhang et al. (23)	China	I: 32 C: 32	I: 7:25 C: 12:20	I: 39.0 ± 11.7 C: 41.0 ± 13.5	I: MA C: Sham MA (shallow needling of non-effective acupuncture)	4 wks	PSQI
Zhao et al. (25)	China	I: 34 C: 32	I: 9:25 C: 8:24	I: 51.67 ± 8.70 C: 50.96 ± 8.46	I: EA C: Sham EA (needling of sham acupoints)	5 wks	PSQI PSG
Zhao et al. (24)	China	I:30 C:30	I: 12:18 C: 14:16	I: 36.8 ± 10.7 C: 38.4 ± 10.8	I: MA C: Sham MA (a blunt end not penetrating the skin at the same acupoints)	8 wks	PSQI
Zhang et al. (26)	China	I:51 C:53	I: 11:40 C: 10:43	I: 52.00 ± 11.86 C: 55.79 ± 12.72	I: MA C: Sham MA (shallow needling of sham acupoints)	4 wks	PSQI
Zhu et al. (27)	China	I:50 C:50	I: 11:39 C: 15:35	I: 44.17 ± 3.31 C: 46.65 ± 3.24	I: MA C: Sham MA (shallow needling of non-effective acupuncture)	4 wks	PSQI

I, Intervention group; C, Control group; EA, Electroacupuncture; MA, Manual acupuncture; wks, weeks; PSQI, Pittsburgh Sleep Quality Index Scale; PSG, Polysomnography.

### 3.3 Risk of bias and quality assessment

#### 3.3.1 Risk of bias assessment

**Figure 2:**
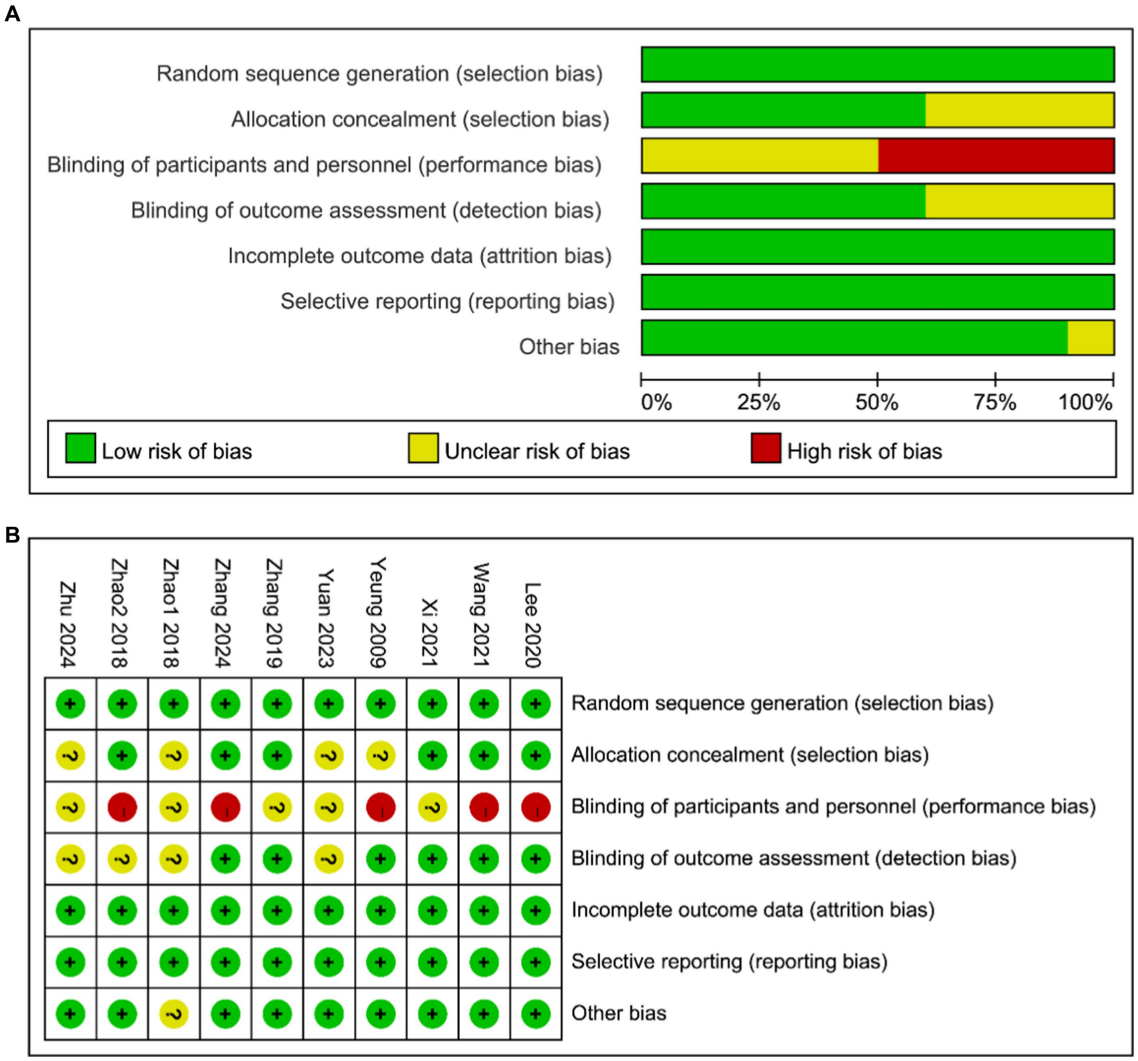
**(A)** Assessment of risk of bias presented as percentages across all included studies. **(B)** Risk of bias summary for each included study.

In terms of sequence generation, three study (20, 26, 27) utilized randomized number tables, while seven studies (12, 13, 21–25) employed software generation (low risk). For allocation concealment, six studies (12, 13, 20, 23, 24, 26) implemented opaque envelopes (low risk), while the remaining studies did not provide sufficient information to assess this criterion (unclear). Regarding blinding of participants and personnel, five studies (12, 13, 21, 24, 26) did not blind therapists (high risk), while the rest did not clearly state whether blinding was performed or not. In terms of blinding of outcome assessment, six studies (12, 13, 20, 21, 23, 26) blinded outcome evaluators (low risk), but for the rest of the studies it was unclear if blinding was conducted. Regarding the completeness of the outcome data, eight studies (12, 13, 20–22, 25–27) provided reasons for participant dropouts or missing data (low risk), whereas two studies (23, 24) had no participant detachment mentioned (low risk). For selective reporting, all studies reported the prespecified outcome indicators (low risk). Regarding other biases, nine studies (12, 13, 20–24, 26, 27) described ethics and conflict of interest (low risk). However, one study (25) failed to provide any information regarding these aspects. Risk assessment results are presented in Figures 2A,B.

#### 3.3.2 Study quality assessment

The quality of acupuncture studies was assessed using the revised STRICTA (2010 version) (see Supplementary File 3). Nine studies used TCM acupuncture, with treatment guided by TCM theories (13, 20–27), and one study used Korean acupuncture, with treatment guided by Korean theories (12). All studies reported the number and names of acupuncture points, and the sensation of Deqi during needling (12, 13, 20–27). All studies involved only two types of needling, manual acupuncture or electroacupuncture, and left the needles in place for 20 or 30 min each time (12, 13, 20–27). Six studies reported the depth of needling (12, 13, 20, 24–26), and four studies did not report the depth of needling (21–23, 27). Ten studies described the parameters of the acupuncture needles and the manufacturers’ information (12, 13, 20–27).

Each study described the specific intervention protocols, duration of each treatment, frequency of weekly treatments, and treatment cycles for both the acupuncture and control groups (12, 13, 20–27).10 studies provided 20–30 min of treatment per session, 2–5 weekly treatments, and treatment cycles of 2–8 weeks (12, 13, 20–27).

Five studies had patients in both groups given sleep hygiene education at the same time (12, 20, 22, 24, 27), and the remaining studies did not provide whether other interventions were performed. Five studies described the background of the acupuncturist (12, 13, 20, 21, 27). Ten studies signed patient informed consent forms (12, 13, 20–27).

### 3.4 Outcomes

#### 3.4.1 Pittsburgh sleep quality index scale

**Figure 3:**
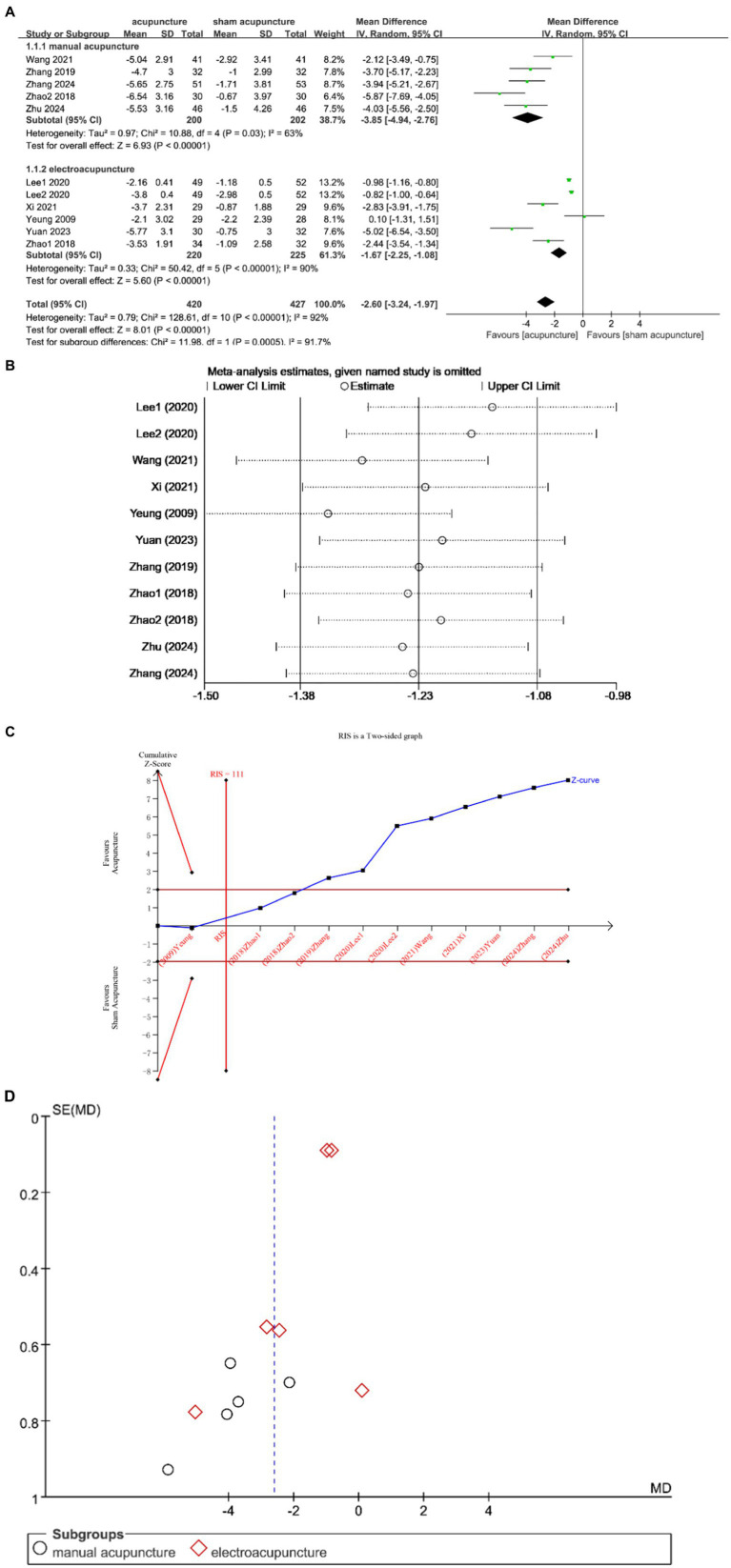
**(A)** Forest plot of PSQI scale scores. **(B)** Sensitivity analysis of PSQI. **(C)** Trial sequential analysis of PSQI. **(D)** A funnel plot of PSQI.

Ten studies (12, 13, 20–27) investigated changes in PSQI before and after treatment, with a total of 847 subjects enrolled. The studies exhibited substantial heterogeneity (I^2^ = 92%, *p* < 0.00001), which was analyzed using random effects modeling. Acupuncture demonstrated statistically significant improvement in patients’ sleep compared to sham acupuncture [MD = −2.60, 95% CI = (−3.24, −1.97), *p* < 0.00001] (Figure 3A). Sensitivity analysis confirmed the stability of the results (Figure 3B). The TSA demonstrated that the cumulative Z-curve had crossed the RIS boundary (RIS = 111), indicating that the sample size was sufficient to conclude that acupuncture was more effective than sham acupuncture in improving PSQI scores (Figure 3C). The funnel plot showed a symmetrical distribution of combined effect sizes from the ten studies on PSQI, suggesting a low likelihood of publication bias (Egger’s test showed t = −0.49, *p* = 0.638 > 0.05) (Figure 3D).

Subgroup analysis according to acupuncture modality showed that manual acupuncture and electroacupuncture were superior to sham acupuncture in improving PSQI scores [MD = −3.85, 95% CI = (−4.94, −2.76), *p* < 0.00001; MD = −1.67, 95% CI = (−2.25, −1.08), *p* < 0.00001] (Figure 3A).

#### 3.4.2 Insomnia severity index

**Figure 4:**
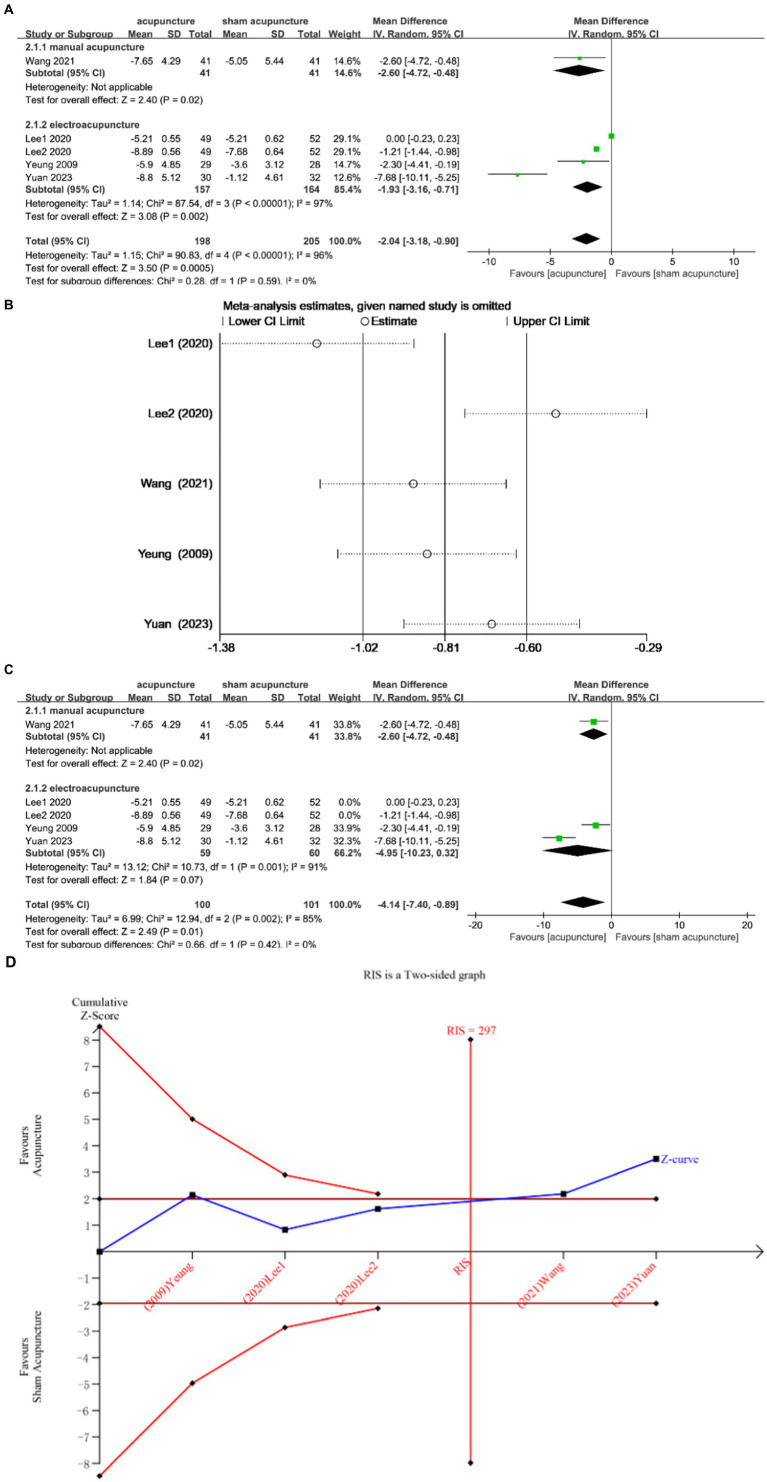
**(A)** Forest plot of ISI scale scores. **(B)** Sensitivity analysis of ISI. **(C)** Sensitivity analysis of ISI. **(D)** Trial sequential analysis of ISI.

Four studies (12, 13, 21, 22) discussed changes in ISI before and after treatment, enrolling 403 subjects, 198 in the experimental group and 205 in the control group. The heterogeneity between studies was large (I^2^ = 96%, *p* < 0.00001) and was analyzed using a random effects model. The results showed that acupuncture was effective in improving patients’ ISI scores compared with sham acupuncture [MD = −2.04, 95% CI = (−3.18, −0.90), *p* = 0.0005] (Figure 4A). Sensitivity analysis showed that the Lee 2020 study had a large impact on the results (Figure 4B), and exclusion showed that the results did not change (Figure 4C), confirming that the results were stable. In the comparison between acupuncture and sham acupuncture, the TSA showed that the cumulative Z curve had crossed the RIS boundary (RIS = 297), indicating that the sample size was sufficient to determine that acupuncture treatment was superior to sham acupuncture in improving ISI (Figure 4D).

Subgroup analysis according to acupuncture modality showed that manual acupuncture and electroacupuncture were superior to sham acupuncture in improving ISI scores [MD = −2.60, 95% CI = (−4.72, −0.48), *p* = 0.02; MD = −1.93, 95% CI = (−3.16, −0.71), *p* = 0.002] (Figure 4A).

#### 3.4.3 Total sleep time

**Figure 5:**
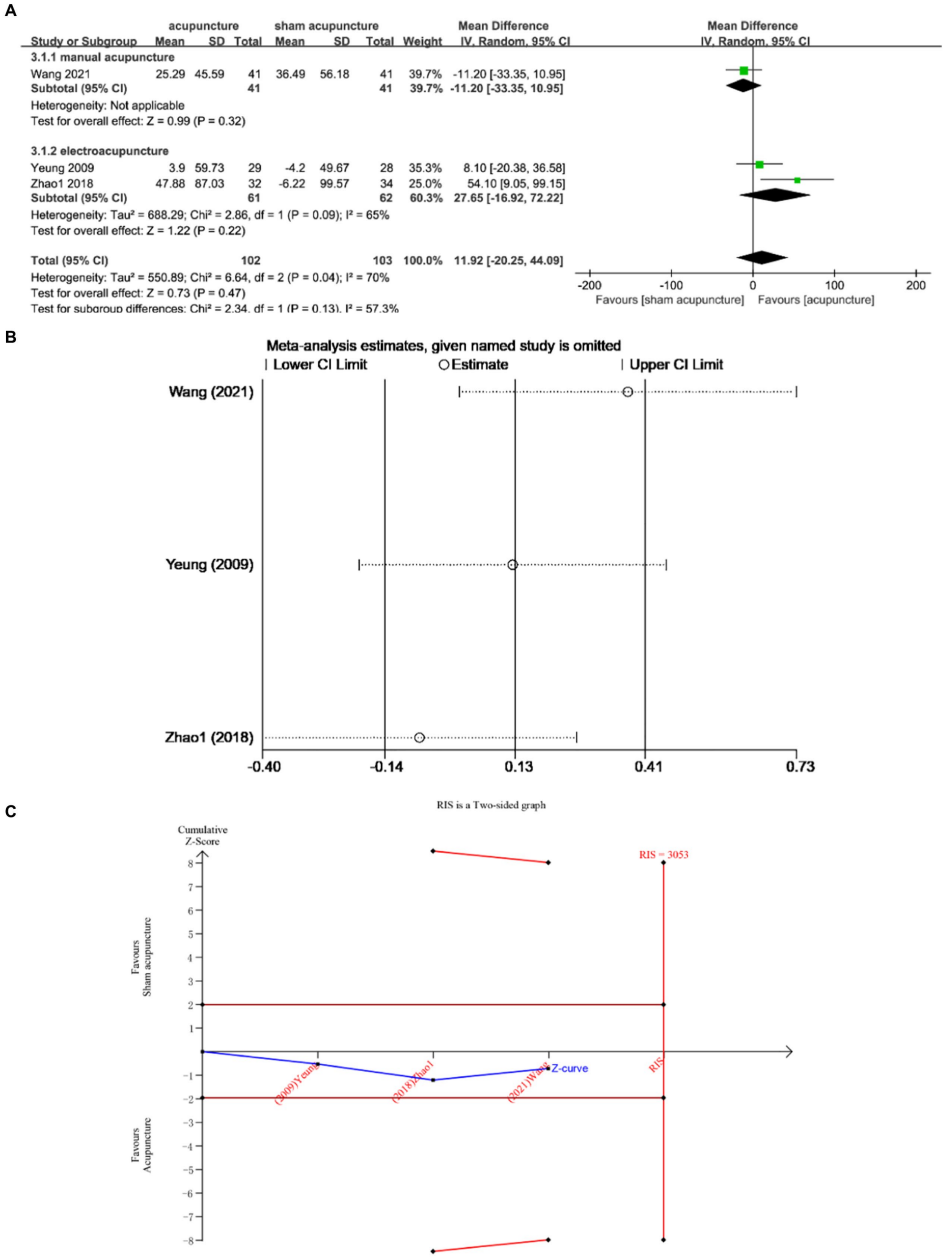
**(A)** Forest Forest plot of TST scores. **(B)** Sensitivity analysis of TST. **(C)** Trial sequential analysis of TST.

Three studies (13, 21, 25) evaluated TST changes before and after treatment, involving a total of 205 subjects. The studies exhibited substantial heterogeneity (I^2^ = 70%, *p* = 0.04), thus a random effects model was employed. No statistically significant difference was observed between the two groups (MD = 11.92, 95% CI = [−20.25, 44.09], *p* = 0.47) (Figure 5A). Sensitivity analysis confirmed the stability of the results (Figure 5B). TSA demonstrated that the cumulative Z curve did not cross the RIS boundary (RIS = 3,053), indicating insufficient sample size to determine whether acupuncture is superior to sham acupuncture in improving TST (Figure 5C).

Subgroup analysis according to acupuncture modality showed that there was no statistically significant difference between manual acupuncture and sham acupuncture [MD = −11.20, 95% CI = (−33.35, 10.95), *p* = 0.32]. Electroacupuncture did not demonstrate superiority over sham acupuncture [MD = 27.65, 95% CI = (−16.92, 72.22), *p* = 0.22] (Figure 5A).

#### 3.4.4 Sleep efficiency

**Figure 6:**
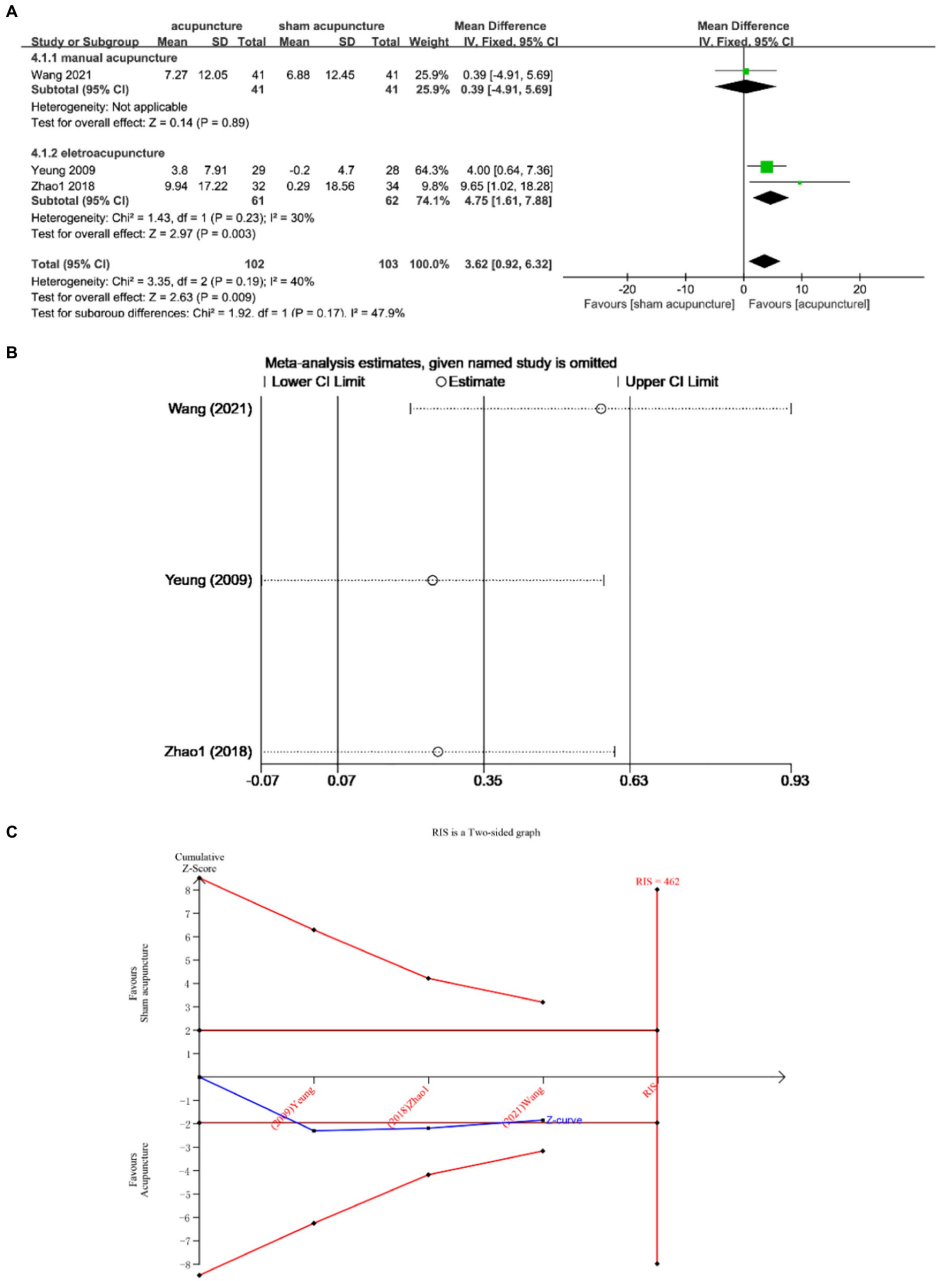
**(A)** Forest plot of SE. **(B)** Sensitivity analysis of SE. **(C)** Trial sequential analysis of SE.

Three studies (13, 21, 25) examined the changes in SE before and after treatment involving a total of 205 subjects. The studies exhibited low heterogeneity (I^2^ = 40%, *p* = 0.19), which was analyzed using fixed effects modeling. The findings demonstrated a statistically significant difference [MD = 3.62, 95% CI = (0.92, 6.32), *p* = 0.009] (Figure 6A). Sensitivity analysis confirmed the stability of the results (Figure 6B). Furthermore, based on TSA analysis, the cumulative Z curve did not cross the RIS boundary (RIS = 462), indicating insufficient sample size to determine whether acupuncture outperformed sham acupuncture in improving TST (Figure 6C).

Subgroup analysis according to acupuncture modality showed that there was no statistically significant difference between manual acupuncture and sham acupuncture [MD = 0.39, 95% CI = (−4.91, 5.69), *p* = 0.89]. Electroacupuncture was superior to sham acupuncture [MD = 4.75, 95% CI = (1.61, 7.88), *p* = 0.003] (Figure 6A).

#### 3.4.5 Wake after sleep onset

**Figure 7:**
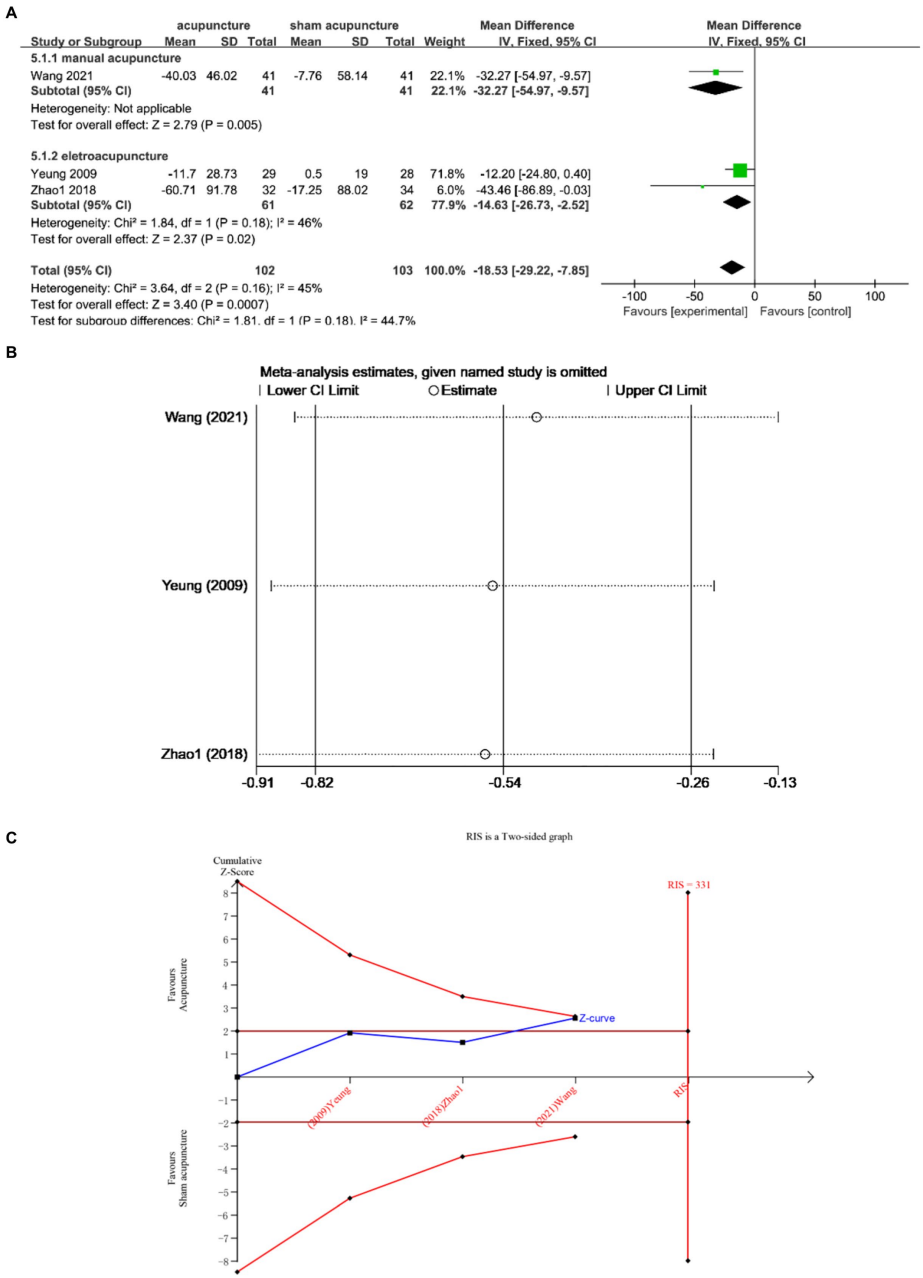
**(A)** Forest plot of WASO. **(B)** Sensitivity analysis of WASO. **(C)** Trial sequential analysis of WASO.

Three studies (13, 21, 25) examined the changes in WASO before and after treatment, involving a total of 205 participants. The studies exhibited low heterogeneity (I^2^ = 45%, *p* = 0.16), which was analyzed using fixed effects modeling. The findings demonstrated a statistically significant difference [MD = −18.53, 95% CI = (−29.22, −7.85), *p* = 0.0007] (Figure 7A). Sensitivity analysis confirmed the stability of the results (Figure 7B). Furthermore, based on TSA analysis, the cumulative Z curve did not cross the RIS boundary (RIS = 331), indicating insufficient sample size to determine whether acupuncture outperformed sham acupuncture in improving WASO (Figure 7C).

Subgroup analysis according to acupuncture modality showed that manual acupuncture and electroacupuncture were superior to sham acupuncture [MD = −32.27, 95% CI = (−54.97, −9.57), *p* = 0.005; MD = −14.63, 95% CI = (−26.73, −2.52), *p* = 0.02] (Figure 7A).

### 3.5 Description of acupuncture regimen

The acupuncture protocol utilized in this study is presented in Table 2, wherein a total of 1–9 major acupoints were selected for each treatment. Specifically, Baihui (GV20), Yintang (EX-HN3), Shenmen (HT7), Sanyinjiao (SP6), and Shenting (DU24) were consistently employed across ≥3 studies.

**Table 2:** Summary of acupuncture treatment.

No.	Study	Acupuncture type	Acupoints	Treatment
1	Lee et al. (12)	EA	Baihui (GV20), Yintang (EX-HN3), Shenmen (HT7), Neiguan (PC6), Jinmen (BL63), Dazhong (KI4)	2–3 times per week for 4 wks; 30 min/session; 10 sessions totally
2	Wang et al. (13)	MA	Shenmen (HT7), Fuliu (KI7)	3 times a week for 3.5 wks; 20 min/session; 10 sessions totally
3	Xi et al. (20)	EA	Baihui (GV20), Shenting (DU24), Yintang (EX-HN3), Shenmen (HT7), Sanyinjiao (SP6)	3 times a week for 4 wks; 30 min/session; 12 sessions totally
4	Yeung et al. (21)	EA	Yintang (EX-HN3), Baihui (GV20), Ear Shenmen, Sishencong (EX-HN1), Anmian (EX)	3 times a week for 3 wks; 30 min/session; 9 sessions totally
5	Yuan et al. (22)	EA	Baihui (GV-20), Yintang (EX-HN3), Shenting (DU24), Shenmen (HT-7), Sanyinjiao (SP6), Taichong (LR3)	3 times a week for 2 wks; 30 min/session; 6 sessions totally
6	Zhang et al. (23)	MA	Shangwan (RN13), Zhongwan (RN12), Xiawan (RN10), Qihai (RN6), Zusanli (ST36), Tianshu (ST25), Neiguan (PC6)	3 times a week for 4 wks; 30 min/session; 12 sessions totally
7	Zhao et al. (25)	EA	Sanyinjiao (SP6)	5 times per week for 5 wks; 30 min/session; 25 sessions totally
8	Zhao et al. (24)	MA	Sishencong (EX-HN1), Baihui (GV20), Shenting (GV24), Benshen (GB13), Taixi (KI3), Shenmen (HT7)	3 times a week for 8 wks; 30 min/session; 24 sessions totally
9	Zhang et al. (26)	MA	Xinshu (BL15), Pishu (BL20), Baihui (GV20), Shenmen (HT7), Zhaohai (KI6), Shenmai (BL62), Anmian (EX-HN18), Sanyinjiao (SP6), Zusanli (ST36)	30 min/session, once every other day; 14 sessions totally
10	Zhu et al. (27)	MA	Baihui (GV20), Shenting (DU24), Sishencong (EX-HN1), Benshen (GB13), Shenmen (HT7), Neiguan (PC6), Sanyinjiao (SP6)	3 times a week for 4 wks; 30 min/session; 12 sessions totally

EA, Electroacupuncture; MA, Manual acupuncture; wks, weeks.

### 3.6 Adverse event reporting

In five studies (13, 20, 21, 24, 27), adverse events related to acupuncture have been reported. Two studies (12, 22) reported no adverse events during the study period. Three studies (23, 25, 26) did not report adverse events.

Wang et al. (13) reported that one subject in the control group withdrew due to foot swelling. It was judged by the study leader that it might not be related to the way of intervention. Some subjects experienced localized subcutaneous bleeding after needle removal, but the bleeding did not last more than 1 min and recovered spontaneously.

Xi et al. (20) reported that no serious adverse reactions such as needle fainting, infection, allergy, etc. occurred in both groups; 2 cases of minor local hematoma in the acupuncture group were not given special treatment and the symptoms disappeared within 1 week.

Yeung et al. (21) reported that both the electroacupuncture group and the control group were well tolerated. Two subjects in the electroacupuncture group developed headache, one developed hand numbness, and one developed hematoma at the acupuncture site; one subject in the control group reported headache, one reported hand numbness, and one reported worsening insomnia. The severity of all adverse events was mild.

Zhao et al. (24) reported 1 minor hematoma after needling in 1 patient in the treatment group who received cold packs and recovered after 2d. No serious adverse events were reported and none withdrew from the trial.

Zhu et al. (27) reported four adverse reactions in the experimental group, including local hematoma, numbness, skin allergy, and dizziness, while three adverse reactions were documented in the control group (two cases of alcohol-induced skin allergy and one case of joint swelling and pain). All adverse events were mild in severity, resolved with appropriate management, and patients continued their treatment.

Overall, all reported related adverse events were mild and recoverable.

## 4 Discussion

### 4.1 Summary of evidence and analysis

Previously published Meta-analyses showed that acupuncture was superior to sham acupuncture in improving subjective sleep in patients with insomnia, but yielded conflicting conclusions about improving objective sleep. A study showed that acupuncture was superior to sham acupuncture in improving TST, WASO, and SE (15). Another study showed that there was no statistically significant difference in improvement of TST, WASO and SE with acupuncture compared to sham acupuncture (16). Both studies included different types of insomnia (e.g., perimenopausal insomnia, depressive insomnia, etc.) and did not differentiate between the course of insomnia. The type and duration of insomnia determined the specific protocol of acupuncture, so this may have contributed to the conflicting conclusions. In addition, neither study explored whether the sample sizes included led to stable conclusions. Therefore, this study evaluated acupuncture for the treatment of patients with primary chronic insomnia and explored whether acupuncture was superior to sham acupuncture in improving subjective and objective sleep indices. At the same time, the methodology of trial sequential analysis was applied to explore whether the sample size of the current study allowed stable conclusions to be drawn.

A total of ten studies meeting strict inclusion and exclusion criteria were selected for qualitative and quantitative analysis. The findings indicate that acupuncture is a feasible treatment option for CID, with no serious adverse events reported. The PSQI is a scale used by patients to assess their subjective sleep quality and the ISI is a scale used to assess the severity of insomnia in patients (28, 29). Meta-analysis results demonstrate that acupuncture significantly improves sleep quality and the severity of insomnia compared to sham acupuncture. Sequential analysis confirms the adequacy of the sample size in obtaining more stable results. Subgroup analysis showed that the efficacy of manual acupuncture and electroacupuncture was better than that of the sham acupuncture group.

In patients with CID, there is a prevalent discrepancy between subjective and objective sleep duration, such as an underestimation of the total recorded sleep (30). Therefore, it is imperative to analyze data from objective indicators (polysomnography or Actigraphy). The meta-analysis of three studies revealed no statistically significant difference in total sleep time between the two groups. According to the sequential analysis of the trials included in the review, the number of patients enrolled was insufficient to estimate whether acupuncture could significantly improve total sleep time in patients. In terms of sleep efficiency, the results demonstrated a statistically significant disparity compared to sham acupuncture; however, the inclusion of an insufficient sample size did not sufficiently establish the effectiveness of acupuncture in improving patients’ sleep efficiency. In terms of wake after sleep onset, acupuncture and sham acupuncture exhibited statistically significant differences; however, the included sample size was insufficient to draw stable conclusions. This shows that there is still a need to conduct clinical trials and expand the sample size of the studies in order to obtain stable conclusions in terms of improving objective sleep indicators. Subgroup analysis according to acupuncture modality showed that the difference between manual acupuncture and sham acupuncture was not statistically significant in improving TST and SE, and acupuncture was superior to sham acupuncture in improving WASO. Electroacupuncture demonstrated superiority over sham stimulation in improving SE and WASO, while no significant difference was observed in TST. This shows that electroacupuncture is more effective, but the sample size included is too small and further studies are still needed.

Publication bias was observed in the included studies, with significant methodological limitations and biases identified. Therefore, more high-quality RCT studies are needed to validate the effectiveness of acupuncture in the treatment of chronic insomnia disorders. In terms of the completeness and quality of the study, most areas were reported in their entirety, with the exception of the acupuncturist’s background, which helped the clinical practitioner to read and understand the article. In addition, this study showed that Baihui (GV20), Yintang (EX-HN3), Shenmen (HT7), Sanyinjiao (SP6), and Shenting (DU24) acupoints were used with high frequency. This is a regular acupoint commonly used in clinical practice for the treatment of insomnia disorders.

Contemporary research has revealed that acupuncture exerts its sleep-enhancing effects through the regulation of neurotransmitters, hormones and cytokines. Notably, acupuncture has been shown to elevate plasma levels of amino acid neurotransmitters such as glutamate and *γ*-aminobutyric acid (GABA), thereby improving sleep quality in individuals with insomnia by enhancing cerebral circulation (31, 32). Recent studies have highlighted the crucial role of inflammatory cytokines in NREM sleep regulation; for instance, IL-1 and TNF-a have been found to promote NREM sleep (33–35). In an animal model of insomnia rats, acupuncture was found to stimulate the production of IL-1β and TNF-*α* while maintaining cytokine balance, thus bolstering the immune system and ameliorating insomnia symptoms (36, 37). Furthermore, acupuncture modulates sleep patterns by influencing interactions among key brain networks including the default network, emotional network, and executive control network. In patients with insomnia, functional connectivity in the blueprint was increased in regions of the sensory cortex and default mode network (38). fMRI analysis indicated a decrease in connectivity between the default mode network and left inferior frontal gyrus after acupuncture treatment—suggesting a potential mechanism underlying its therapeutic efficacy (39). Collectively from numerous investigations conducted thus far, it is evident that acupuncture represents a promising therapeutic option for chronic insomnia disorder.

### 4.2 Strengths and limitations

The strength of this study is that, compared with other meta-analyses of acupuncture for insomnia disorders (15, 16, 40–42), individual assessment of primary CID. To investigate whether acupuncture is effective in improving subjective and objective sleep indices in patients with chronic insomnia compared with sham acupuncture. In addition, a trial sequential analysis was conducted to explore whether the sample size included was sufficient to support the drawing of stable conclusions. This informs the design of future trials of clinical acupuncture for chronic insomnia disorders.

The limitation of this study lies in the high inter-study heterogeneity, potentially attributed to variations in acupuncture interventions, acupuncture sessions, number of needles, and point selection. Future clinical trials can determine the quantitative-effectiveness relationship between acupuncture in treating CID by examining acupuncture interventions, acupuncture sessions, and number of acupuncture sessions as well as acupoints and combinations. In terms of objective indicators, the small sample size does not yet allow us to derive the effectiveness of acupuncture for CID.

## 5 Conclusion

Compared with sham acupuncture, acupuncture can effectively improve subjective sleep in patients with CID, but whether it can improve objective sleep indicators still needs to be verified by future large-sample, high-quality randomized controlled trials.

## Data Availability

The original contributions presented in the study are included in the article/Supplementary material, further inquiries can be directed to the corresponding authors.
